# Fatty Acid Synthase Inhibitors Induce Apoptosis in Non-Tumorigenic Melan-A Cells Associated with Inhibition of Mitochondrial Respiration

**DOI:** 10.1371/journal.pone.0101060

**Published:** 2014-06-25

**Authors:** Franco A. Rossato, Karina G. Zecchin, Paolo G. La Guardia, Rose M. Ortega, Luciane C. Alberici, Rute A. P. Costa, Rodrigo R. Catharino, Edgard Graner, Roger F. Castilho, Aníbal E. Vercesi

**Affiliations:** 1 Departamento de Patologia Clínica, Faculdade de Ciências Médicas, Universidade Estadual de Campinas (UNICAMP), Campinas, SP, Brazil; 2 Departamento de Diagnóstico Oral, Faculdade de Odontologia de Piracicaba, Universidade Estadual de Campinas (UNICAMP), Piracicaba, SP, Brazil; 3 Departamento de Química e Física, Faculdade de Ciências Farmacêuticas de Ribeirão Preto, Universidade de São Paulo (USP), Ribeirão Preto, SP, Brazil; University of Illinois at Chicago, United States of America

## Abstract

The metabolic enzyme fatty acid synthase (FASN) is responsible for the endogenous synthesis of palmitate, a saturated long-chain fatty acid. In contrast to most normal tissues, a variety of human cancers overexpress FASN. One such cancer is cutaneous melanoma, in which the level of FASN expression is associated with tumor invasion and poor prognosis. We previously reported that two FASN inhibitors, cerulenin and orlistat, induce apoptosis in B16-F10 mouse melanoma cells *via* the intrinsic apoptosis pathway. Here, we investigated the effects of these inhibitors on non-tumorigenic melan-a cells. Cerulenin and orlistat treatments were found to induce apoptosis and decrease cell proliferation, in addition to inducing the release of mitochondrial cytochrome c and activating caspases-9 and -3. Transfection with FASN siRNA did not result in apoptosis. Mass spectrometry analysis demonstrated that treatment with the FASN inhibitors did not alter either the mitochondrial free fatty acid content or composition. This result suggests that cerulenin- and orlistat-induced apoptosis events are independent of FASN inhibition. Analysis of the energy-linked functions of melan-a mitochondria demonstrated the inhibition of respiration, followed by a significant decrease in mitochondrial membrane potential (ΔΨm) and the stimulation of superoxide anion generation. The inhibition of NADH-linked substrate oxidation was approximately 40% and 61% for cerulenin and orlistat treatments, respectively, and the inhibition of succinate oxidation was approximately 46% and 52%, respectively. In contrast, no significant inhibition occurred when respiration was supported by the complex IV substrate *N*,*N*,*N′*,*N′*-tetramethyl-*p*-phenylenediamine (TMPD). The protection conferred by the free radical scavenger N-acetyl-cysteine indicates that the FASN inhibitors induced apoptosis through an oxidative stress-associated mechanism. In combination, the present results demonstrate that cerulenin and orlistat induce apoptosis in non-tumorigenic cells *via* mitochondrial dysfunction, independent of FASN inhibition.

## Introduction

The metabolic enzyme fatty acid synthase (FASN) is responsible for the production of saturated fatty acids, such as palmitate, through the condensation of acetyl-CoA and malonyl-CoA [1]–[7]. FASN products are used in the formation of cell membranes [8] and are responsible for a significant number of functions in the body, acting primarily as intracellular messengers and energy stores [9]. In most normal tissues, the expression and activity of FASN are low or absent; exceptions include instances where lipogenesis is necessary, such as in the liver, adipose tissue, breast tissue during lactation, endometrium during the proliferative phase and the lungs of newborns [2], [3], [10], [11]. In contrast, high FASN activity is found in several neoplasias that occur in breast, ovarian, prostate, thyroid, lung, stomach, pancreas, colon, esophagus, mouth and bladder tissues, as well as soft tissue sarcomas and melanoma [10], [12]–[33]. Further, increased FASN expression in malignant tumors is associated with a poor prognosis [4], [13], [14], [16], [17], [21], [24], [28], [29], [33]–[38].

FASN inhibition reduces cell proliferation and induces apoptosis *in vitro* and decreases the size of prostate, ovarian and breast cancer xenografts [39]–[41]. The biological mechanisms responsible for FASN inhibition-induced apoptosis remain unclear. The extrinsic apoptosis pathway, which is triggered by death domains, was described after siRNA silencing of FASN in breast cancer cells caused the accumulation of malonyl-CoA and ceramide [42], [43]. Mitochondrial involvement in apoptosis, as evidenced by increased levels of the pro-apoptotic protein Bax and the release of cytochrome c, has been found in several tumor cell lines, including neuroblastoma, melanoma, colon carcinoma, breast cancer and skin carcinoma, following pharmacological FASN inhibition [37], [44]. Despite the fact that the expression of a dominant-negative mutant p53 increased the sensitivity of colon carcinoma cells to FASN inhibitors [45], FASN inhibition-induced apoptosis was described as a p53-independent process [44]. We recently showed that the inhibition of FASN activity with orlistat significantly impaired lipid synthesis, reduced proliferation and promoted apoptosis in the mouse metastatic melanoma cell line B16-F10 [46], [47]; additionally, similar treatment reduced experimental metastases and angiogenesis in B16-F10 melanomas [48]. We showed that FASN inhibition activates the intrinsic apoptotic pathway, as evidenced by the release of cytochrome c and the activation of caspases-9 and -3; this activation is preceded by increased production of reactive oxygen species and elevated cytosolic calcium concentrations in these melanoma cells [47]. Orlistat treatment of B16-F10 cells also resulted in significant changes in the mitochondrial free fatty acid (FFA) composition, as demonstrated by electrospray ionization mass spectrometry (ESI-MS) [49].

Although several studies suggest that normal cells are more resistant to the cytotoxic action of FASN inhibitors [40], [43], [50]–[52], cerulenin and orlistat significantly reduced the proliferation of normal gingival fibroblasts and endothelial cells [26], [53], [54]. Here, we show that similar to B16-F10 cells, non-tumorigenic melan-a cells exhibit reduced proliferation and undergo apoptosis through the release of cytochrome c and the activation of caspases-9 and -3 when treated with FASN inhibitors. The effect of these FASN inhibitors on the non-tumorigenic cell line used here involves the inhibition of mitochondrial respiration but does not alter the FFA content of these cells.

## Materials and Methods

### Cell Culture and Reagents

Melan-a cells, the first known line of non-tumorigenic mouse melanocytes and a normal counterpart to melanoma cells [55], were obtained from Prof^a^. Miriam Galvonas Jasiulionis (Universidade Federal de São Paulo, São Paulo, Brazil) and were cultured in RPMI-1640 medium (Vitrocell, Brazil) supplemented with 5% fetal bovine serum (Vitrocell), 200 nM 12*-o-*tetradecanoyl phorbol*-*13*-*acetate (TPA, Sigma-Aldrich, St. Louis, MO, USA), 100 mg/ml gentamycin (Vitrocell), 100 IU/ml penicillin (Vitrocell) and 100 mg/ml streptomycin (Vitrocell) at 37°C in a humidified atmosphere with 5% CO_2_. To block FASN activity, either cerulenin (Sigma-Aldrich, USA) or orlistat (Roche, Switzerland) was added to the culture medium at the concentration specified in the figure legends. The *IC*_50_ for β-keto-acyl-ACP synthase inhibition by cerulenin is 1.5 µM, and the *K*_i_ for FASN inhibition by orlistat is 0.30±0.09 µM [56]. Orlistat was extracted from Xenical capsules as previously described [57]. The equivalent concentrations of cerulenin and orlistat solvents, 0.025% DMSO and 0.012% ethanol (EtOH), respectively, were present under control conditions. Cells treated with cerulenin showed faster degeneration than cells treated with orlistat; for this reason different treatment durations were used for cerulenin (6–24 h) and orlistat (24–48 h).

The non-tumorigenic HaCaT cell line, which was derived from human keratinocytes, was purchased from Cell Line Service (CLS, Heidelberg, Germany). HaCaT cells were grown in a high-glucose DMEM culture medium (Vitrocell) supplemented with 10% fetal bovine serum and antibiotics, in the same manner as that described for the melan-a cells.

### Determination of Cell Viability and Proliferation

To determine the antiproliferative and cytotoxic effects of cerulenin and orlistat, the cells were stained with 0.1% trypan blue and then counted in a Neubauer chamber, as previously described [47]. Cell viability was determined by excluding the stained cells, as well as by using a 3-(4,5-dimethylthiazol-2-yl)-2,5-diphenyltetrazolium bromide assay (MTT, Sigma). Briefly, melan-a cells were plated in 6-well culture plates (2.5–6.5×10^4^ cells per well), and after 24 h, the medium was replaced with fresh medium that contained the FASN inhibitors. After an additional 24 or 48 h, the cells were incubated with 2.5 mg/ml MTT for 4 h at 37°C in a 5% CO_2_ incubator. Then, the medium was removed, and 1 ml of absolute ethanol was added to each well for complete solubilization of the generated formazan. The contents were subsequently transferred to 96-well plates, and the absorbance was determined at 540 nm with the aid of a microplate reader (Bio-Rad, USA). In this work, cell viability is expressed as the percentage of viable cells relative to the controls.

### Analysis of Cell Death and Cell Cycle

The samples were analyzed in a FACSCalibur flow cytometer (BD Biosciences, Franklin Lakes, NJ, USA) equipped with an argon laser and Cell-Quest software (version 4.1). Between seven and ten thousand events were acquired per sample. Melan-a populations were identified based on their light-scattering characteristics by enclosing the samples in electronic gates and analyzing for the intensity of the fluorescent probe signal.

For the cell death analysis, melan-a cells (10^6^) were washed with PBS and resuspended in binding buffer (10 mM HEPES pH 7.4, 150 mM NaCl, 5 mM KCl, 1 mM MgCl_2_ and 1.8 mM CaCl_2_) containing annexin V-FITC (1∶500, Invitrogen, USA) and 7-AAD (20 mg/ml, 7-amino-actinomycin D, Molecular Probes, USA), as previously described [47]. Cell apoptosis was quantified by flow cytometry as the number of annexin V-FITC-positive and 7-AAD-negative cells, and necrosis was quantified as the number of 7-AAD-positive and annexin V-FITC-negative cells, both divided by the total number of cells.

Cell cycle analyses were performed as previously described [46], [47]. Melan-a cells were seeded in 6-well culture plates (2.5–6.5×10^4^ cells). After 24 h, the medium was replaced with serum-free medium, and the cells were incubated for an additional 24 h. The medium was replaced with fresh medium containing serum and the respective FASN inhibitor, and the cells were incubated for an additional 24 or 48 h, then harvested and fixed in cold 70% ethanol. The cells were then washed in PBS, treated with 10 µg/ml RNAse for 1 h at 37°C and stained with 50 µg/ml propidium iodide (Sigma) for 2 h at 41°C. The distribution of cells in the cell cycle was analyzed by flow cytometry. The cell cycle phases were analyzed using ModFit LT™ (Verity Software House, USA).

### Measurement of ROS

Following treatment with cerulenin or orlistat for 24 or 48 h 10^6^ viable cells were incubated with 5 µM MitoSOX (Molecular Probes) at 37°C for 10 min to detect mitochondrial superoxide production [58]. The ROS levels were analyzed using a spectrofluorometer (Hitachi, model F-4500, Tokyo, Japan) operating at excitation and emission wavelengths of 510 and 580 nm, respectively, with slits widths of 5 and 10 nm, as previously described [47], [59].

### Detection of Caspase-3 Activation

Caspase-3 activation was assessed by incubating 10^6^ cells with FITC-DEVD-FMK (1∶300, Calbiochem, USA) in serum-free medium for 40 min at 37°C in a humidified atmosphere with 5% CO_2_. After a wash step was performed according to the manufacturer’s instructions, the cells were resuspended in the same medium and analyzed by flow cytometry, as previously described [46].

### Detection of Caspases-9 and -8 Activities

Cells (3×10^6^ cells) were resuspended in 0.2 ml of chilled lysis buffer (20 mM HEPES pH 7.5, 10 mM KCl, 250 mM sucrose, 2 mM MgCl and 1 mM EDTA) containing 0.5 mM DTT. The cell suspensions were sonicated (Mosonix Sonicator S-3000, New Highway Farmingdale, USA) and frozen at −80°C. The cell lysates were thawed and centrifuged at 15,000 *g* for 30 min, and the supernatants were added to 0.2 ml of reaction buffer (25 mM HEPES pH 7.5, 10% sucrose and 0.1% CHAPS) containing 10 mM DTT. The reactions were initiated by the addition of the caspase-8 or −9 substrates, 0.1 mM Ac-LETD-AFC (Sigma) or 0.2 mM LEHD-pnitroanilide (Calbiochem), respectively, and were incubated for 1.5 h at 37°C, as previously described [47]. Caspase-8 activity was determined by measuring the fluorescence of free AFC using a Hitachi F4500 spectrofluorometer (Hitachi High-Tech, Japan), with excitation and emission wavelengths of 400 and 505 nm, respectively, and slit widths of 5.0 nm. Melan-a cells treated for 20 h with 1.25 µg/ml cycloheximide (Sigma) and 10 nM tumor necrosis factor alpha (TNFα, Peprotech, USA) were used as positive controls. Caspase-9 activity was determined by measuring the absorbance of free p-nitroanilide using a Varian Cary 50 spectrophotometer (Biocompare, USA) at 405 nm.

### Detection of Cytochrome c Release

After the cells were treated with cerulenin or orlistat for 12 or 24 h, the release of mitochondrial cytochrome c was detected by flow cytometry [60]. Briefly, 10^6^ cells were washed with PBS, resuspended in 1 ml of mitochondrial medium (125 mM sucrose, 65 mM KCl, 10 mM HEPES buffer pH 7.2, 0.5 mM EGTA, 1 mM MgCl_2_ and 2 mM KH_2_PO_4_) supplemented with 1% mix of protease inhibitors and 1 mM phenylmethylsulfonyl fluoride and then permeabilized with 0.0001% digitonin. Pellets were resuspended in 0.5 ml of 4% paraformaldehyde in PBS and incubated for 20 min at room temperature. After two washes with PBS, the cells were incubated in 0.5 ml of labeling medium (2% fetal bovine serum, 0.2% sodium azide and 0.5% Triton X-100 in PBS) for 15 min, centrifuged at 3,000 *g* for 5 min and then incubated with an anti-cytochrome c antibody (1∶500, 6 H2.B4, Promega, USA) at 4°C for 1 h. The cells were then washed twice in the same medium and incubated with an anti-mouse-FITC antibody (1∶200, Vector Laboratories, USA) at 4°C for 1 h. The cells were washed once more as described above, resuspended in PBS and analyzed by flow cytometry as described elsewhere [60].

### Assessment of Mitochondrial Membrane Potential (ΔΨm)

The ΔΨm in the digitonin-permeabilized melan-a cells was estimated by changes in Safranin O fluorescence [61], as recorded using a spectrofluorometer (Hitachi, model F-4500, Tokyo, Japan) operated at excitation and emission wavelengths of 495 and 586 nm, respectively, with slits widths of 5 nm. Melan-a cells were treated with 22 µM cerulenin for 6 h or with 30 µM orlistat for 24 h. Approximately 2×10^6^ viable cells were permeabilized with 15 µM digitonin in 2 ml of reaction medium containing 125 mM sucrose, 65 mM KCl, 10 mM HEPES, 1 mM MgCl_2_, 65 mM Tris-HCl (pH 7.2), 2.5 mM Na_2_HPO_4_, 50 µM EGTA, 5 mM succinate, 0.01% BSA and 5 µM Safranin O; then, the reactions were incubated at 37°C while stirring [59], [62]–[64].

### Cellular Respiration

Following either 24 h of treatment with 22 µM cerulenin or 48 h of treatment with 30 µM orlistat, the consumption of oxygen by the melan-a cells was measured using a closed-chamber high-resolution respirometry Oroboros (Innsbruck, Austria) equipped with a magnetic stirrer and temperature control set at 37°C [65]–[67]. Approximately 2×10^6^ viable cells were added to 2 ml of reaction medium containing 125 mM sucrose, 65 mM KCl, 10 mM HEPES, 2.0 mM K_2_HPO_4_, 1.0 mM MgCl_2_ (pH 7.2); 50 µM EGTA, 0.01% BSA and NADH-linked substrates (2.0 mM malate, 1.0 mM α-ketoglutarate, 1.0 mM pyruvate and 1.0 mM glutamate). Then, the melan-a cells were permeabilized by the addition of 15 µM digitonin, and the oxidative phosphorylation and mitochondrial respiratory activity were analyzed by the sequential addition of 300 µM ADP, 2 µg/ml oligomycin, 100 nM carbonylcyanide p-trifluoromethoxyphenylhydrazone (FCCP), 5 mM succinate, 0.5 µM antimycin and 200 µM N,N,N′,N′-tetramethyl-p-phenylenediamine (TMPD) with 2 mM ascorbate. The data were determined using the device software.

### Citrate Synthase Activity

Citrate synthase activity in the cell suspension was analyzed by spectrophotometry based on the conversion of oxaloacetate and acetyl-CoA to citrate and SH-CoA. This reaction is catalyzed by citrate synthase and was monitored by measuring the colorimetric product thionitrobenzoic acid [47]. Cytosolic fractions were incubated at 37°C in a buffer containing 50 mM Tris–HCl (pH 8.0), 0.1% Triton X-100, 250 µM oxaloacetate, 50 µM acetyl-CoA and 100 µM 5,50-dithiobis-(2-nitrobenzoic acid). The increase in absorbance at 412 nm was recorded over 8 min.

### RNA Interference (RNAi)-Mediated Silencing of FASN Expression

Twenty-five-mer RNA molecules were chemically synthesized, annealed and purified by the manufacturer (Stealth RNAi, Invitrogen). Three sequences targeting *Mus musculus* FASN (NM_00798) were used, corresponding to nucleotides 940–964 (50-CAATGATGGCCAACCGGCTCTCTTT-30), 3408–3432 (50-TGGGAAGACCCGAACTCCAAGTTAT-30) and 5841–5865 (50-CCTCTGGGCATGGCTATCTTCTTGA-30), as previously described [46]. Melan-a cells grown to 50% confluence were transfected with 200 nM of a mixture containing equal parts of the FASN siRNAs using a liposome method according to the manufacturer’s instructions (Lipofectamine 2000, 2 µg/ml, Invitrogen). Negative control cells were transfected with equimolar concentrations of a nonspecific control oligo (Stealth RNAi Negative Control Duplexes, Medium GC Duplex, Invitrogen). Transfections were performed in 35-mm^2^ dishes, and after 48 h, the cells were collected to assess FASN knockdown and to detect cell death. FASN knockdown was confirmed by Western blot analysis using approximately 40 µg of the protein lysates and antibodies against FASN (BD Biosciences, 1∶3000) or beta-actin (AC-15, Sigma, 1∶40 000) as a loading control. The reactions were developed with an enhanced chemiluminescence detection system (ECL detection kit, Amersham Pharmacia Biotech, USA) according to the manufacturer’s instructions.

### Electrospray Ionization Mass Spectrometry (ESI-MS)

ESI-MS was used to analyze the melan-a mitochondria, as previously described [49], with few modifications. After the mitochondria were extracted, the total protein content was quantified using the Bradford method. Lipid extraction was performed as described by Bligh and Dyer (1959) [68]. The mitochondria-containing pellets were resuspended in 0.1 ml of ultrapure H_2_O, and 0.5 ml of a solution of methanol/toluene (7∶3 v/v) and 0.05 ml of a methanol solution of ammonia (0.1% v/v) were added to each sample. This diluted solution was then directly infused according to the following protocol. A total of 16 samples was analyzed, 4 from the control and 4 from the treated cells for each FASN inhibitor.

Lipid analyses were performed in negative mode using an ESI Q-TOF Premier (Waters) coupled with a nanoelectrospray source introduced *via* direct injection, performed at a flow rate of 10 µL/min and using a Harvard Apparatus pump. The nanoelectrospray voltage was set to 2.5 kV; the cone voltage, to 40 V; the source temperature, to 120°C; the desolvatation temperature, to 200°C; and the collision energy, to 10 V. The instrument was operated in MS continuum mode, and data were acquired from m/z 50–1.000 with a scan rate of 1 s and an interscan delay of 0.1 s. The data were analyzed using the Masslynx 4.1 software package. The spectra were accumulated over 6 s in the region with flow rate stability. The spectra were smoothed (2×3 channels, Savitzky Golay smooth), and the mass centroid values were obtained using 80% of the peak top and the minimum peak width at half-height of 4 channels. Principal component analysis was performed using the MetaboAnalyst software. Data were autoscaled for principal component analysis (PCA) and partial least square discriminant analysis (PLS-DA). The analyses were performed using the MetaboAnalyst online platform [69], [70], supported by the use of the Piroeutte (v. 4.0, Infometrix, Inc.) software.

### Statistical Analysis

The results from at least three independent experiments, each performed in duplicate or triplicate, are displayed as the mean ± s.e.m. Comparisons between the groups were performed using One-Way Analysis of Variance with Tukey’s post-hoc analysis. The level of significance was set at *p*<0.05. All data were analyzed using SigmaStat software, version 3.5 (Systat Software, USA).

## Results

### FASN inhibitors decrease melan-a cell viability and proliferation and induce cell death in a dose-dependent manner

**Figure 1:**
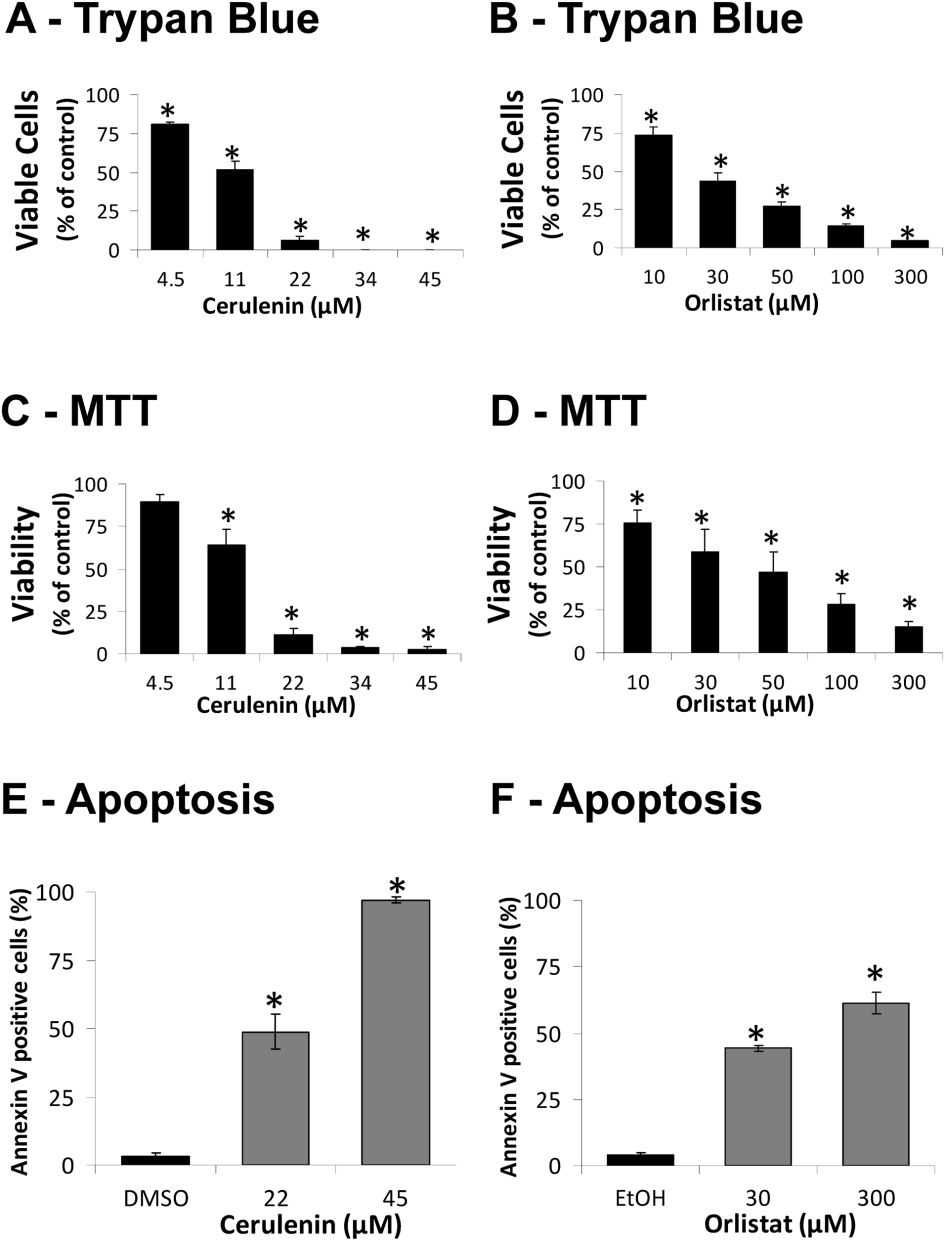
Cerulenin and orlistat reduce cell viability and induce apoptosis in the melan-a cell line. Melan-a cells were treated with increasing concentrations of cerulenin or orlistat for 24 or 48 h, respectively; cell viability was determined using trypan blue (**A** and **B**) or MTT assays (**C** and **D**), and apoptosis was determined by flow cytometry after Annexin V staining (**E** and **F**). The values represent the mean ± s.e.m of at least three independent experiments. *Significantly different from the respective control at *p*<0.05.

The viability of melan-a cells was significantly reduced after treatment with cerulenin or orlistat (**Figure 1**). The decrease in cell viability estimated by the trypan blue or MTT (Panels A–D) assays was higher than the apoptotic rate (Panels E–F), possibly due to the higher sensitivity of the test used to estimate cell viability [71]. Death in the melan-a cell line occurred mainly by apoptosis, as we previously demonstrated for the B16-F10 melanoma cells [47], while the necrosis rates remained unchanged by these treatments (DMSO: 2.4±0.5%, 22 µM cerulenin: 2.4±0.5%; EtOH: 1.5±0.8%; 30 µM orlistat: 2.4±0.4%). No significant effect of the vehicles (EtOH and DMSO) on cell viability and apoptosis rate was observed (results not shown).

**Figure 2:**
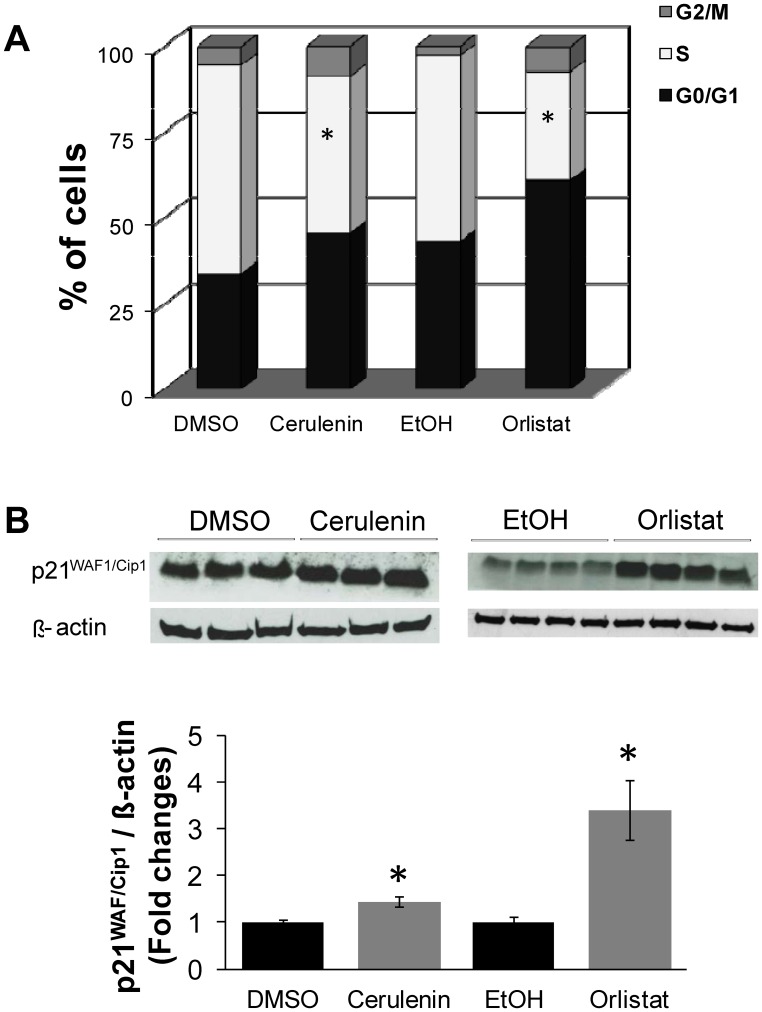
FASN inhibitors blocked cell cycle progression in non-tumorigenic cells. Melan-a cells were treated with 22 µM cerulenin or 30 µM orlistat for 24 or 36 h, respectively. Then, the percentage of cells in each phase of the cell cycle was determined by flow cytometry after PI staining (**A**). Western blot analysis of protein extracts prepared from cerulenin- and orlistat-treated melan-a cells revealed the accumulation of the p21WAF1/Cip1 tumor suppressor protein (**B**). The data were normalized using beta-actin as a loading control. The values represent the mean ± s.e.m of at least five independent experiments. *Significantly different from the respective control at *p*<0.05.

Cell cycle analysis was performed to verify the effects of both FASN inhibitors on cell proliferation. After 24 h of serum starvation, approximately 80% of the melan-a cells were in the G0/G1 phase (*data not shown*). The cerulenin- and orlistat-treated melan-a cells demonstrated reductions of 25% and 42% in S phase, respectively, when compared to the controls (**Figure 2 A**). Cell cycle arrest was confirmed by increased levels of the p21^WAF1/Cip1^ tumor suppressor protein, as shown by Western blot analysis (**Figure 2 B** . The treatment of melan-a cells with cerulenin and orlistat increased p21^WAF1/Cip1^ levels by 1.4- and 3.4-fold, respectively, compared to the control cells.

The FASN inhibitors were also tested in another non-tumorigenic cell line, HaCaT, which is derived from normal keratinocytes. Similar to the melan-a cells, cerulenin and orlistat reduced the viability and proliferation of HaCaT and induced apoptosis (**Figure S1**). The HaCaT cells were more resistant to apoptosis than the melan-a cells, especially when treated with cerulenin while the necrosis rates remained unchanged by these treatments (DMSO: 0.20±0.08%, 45 µM cerulenin: 1.97±0.27%; EtOH: 0.20±0.06%; 300 µM orlistat: 1.67±0.20%). The HaCaT cells also showed lower levels of FASN protein when compared to the melan-a cells, as verified by Western blot analysis (**Figure S2**). Cell cycle analysis of the HaCaT cells after treatment with the FASN inhibitors revealed a significant degree of cell cycle arrest. Cerulenin and orlistat treatment increased the number of cells in G0/G1 phase by 3- and 6-fold, respectively (**Figure S3 A**). Treatment with the FASN inhibitors also increased the p21^WAF/Cip^ protein content by 1.3- or 35-fold when compared to the respective controls (**Figure S3 B**).

### Mitochondrial dysfunction participates in melan-a cell death induced by FASN inhibitors

**Figure 3:**
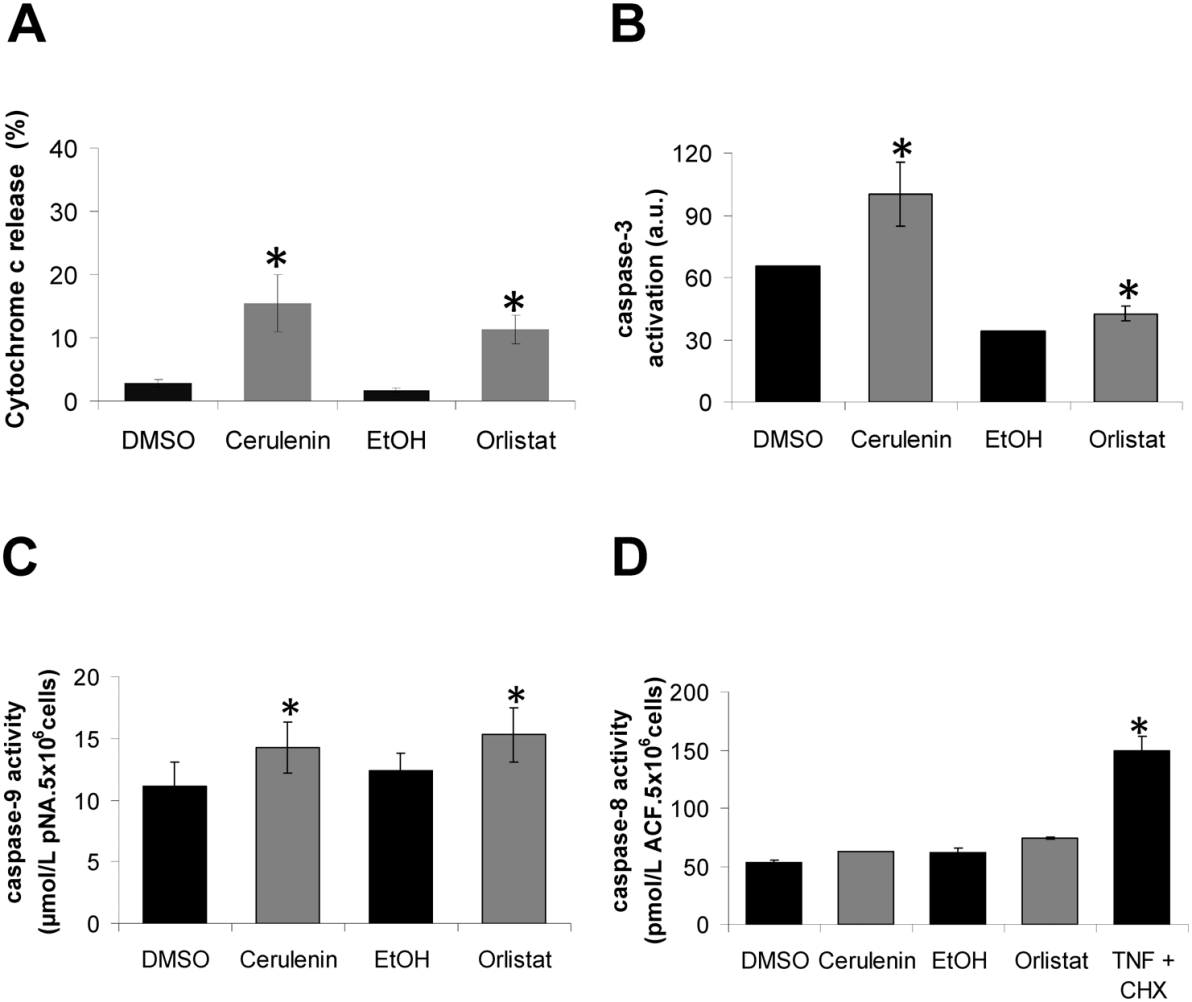
Treatment of melan-a cells with FASN inhibitors leads to the release of mitochondrial cytochrome c and the activation of caspases-3 and -9 but not -8. Melan-a cells were treated with 22 µM cerulenin or 30 µM orlistat for 12 or 24 h, respectively; then, the release of cytochrome c was determined by flow cytometry (**A**). The cells were also treated with cerulenin or orlistat under the same conditions, and the activation of caspase-3 was estimated using FITC-DEVD-FMK (**B**). The activities of caspase-9 and -8 (**C** and **D**) were determined as described in Material and Methods. The values represent the mean ± s.e.m of at least three independent experiments. *Significantly different from the respective control at *p*<0.05.

Recently, we demonstrated the release of mitochondrial cytochrome c in the FASN inhibition-induced apoptosis of B16-F10 melanoma tumor cells [47]. Because FASN inhibitors also induced apoptosis in the melan-a cells (**Figures 1 E and F**), the non-tumorigenic melanoma cell counterpart [55], the percentage of cytochrome c released was also determined in these cells. Cerulenin and orlistat treatment for 12 and 24 h culminated in the release of cytochrome c in the melan-a cells (16% and 12%, respectively) (**Figure 3 A**), and this finding was accompanied by the activation of caspase-3 (52% and 24%, respectively) (**Figure 3 B**) and caspase-9 (28% and 24%, respectively) (**Figure 3 C**). No significant differences were found in caspase-8 activity (**Figure 3 D**). Pre-treatment with cyclosporin A, which is a classic inhibitor of mitochondrial permeability transition, did not protect the melan-a cells from cerulenin- or orlistat-induced apoptosis (**Figure S4 A**). Apoptosis in the melan-a cells was also shown to be independent of p53 activation, as pre-treatment with *pifithrin-alpha* (PFT), which is a known synthetic inhibitor of p53 [72], did not prevent FASN inhibitor-induced cell death (**Figure S4 B**).

**Figure 4:**
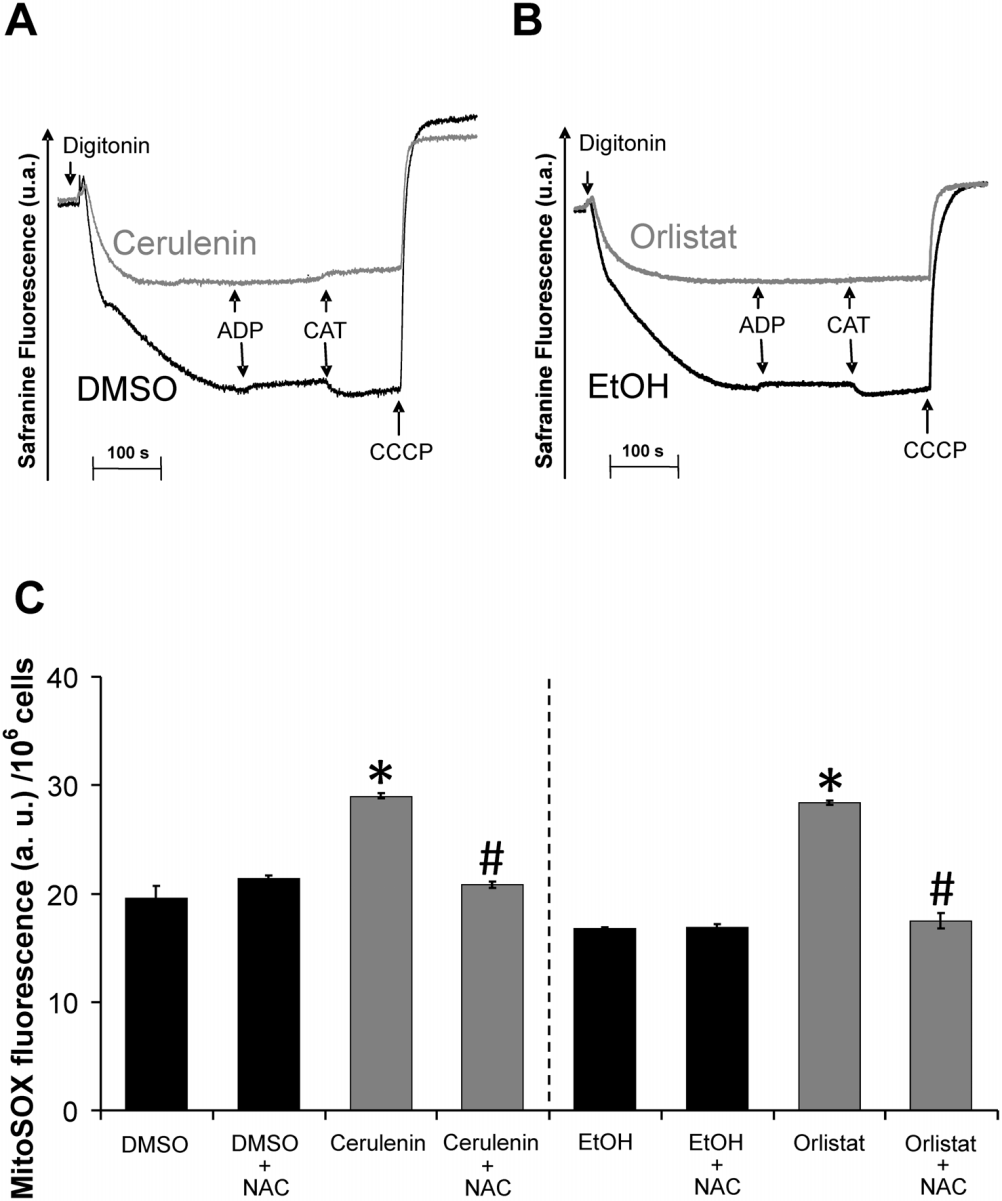
FASN inhibitors result in decreased ΔΨm and increased superoxide production in melan-a cells. Melan-a cells were treated with 22 µM cerulenin or 30 µM orlistat for 6 or 24 h, respectively; then, approximately 2×10^6^ viable cells/ml were permeabilized with 15 µM digitonin. ΔΨm was estimated by Safranin fluorescence. The arrows indicate the addition of 15 µM digitonin, 100 µM ADP, 5 µM carboxyatractyloside (CAT) and 1 µM CCCP (**A** and **B**, representative of at least three independent experiments). Melan-a cells were also treated with 22 µM cerulenin or 30 µM orlistat for 24 or 48 h, respectively and also incubated in the presence or absent of NAC; the cells were then washed and probed with 5 µM MitoSOX (**C**). The values represent the mean ± s.e.m of four independent experiments. *Significantly different from the respective control at p<0.05. #Significantly different from the respective condition in the absence of NAC at *p*<0.05.

**Figure 5:**
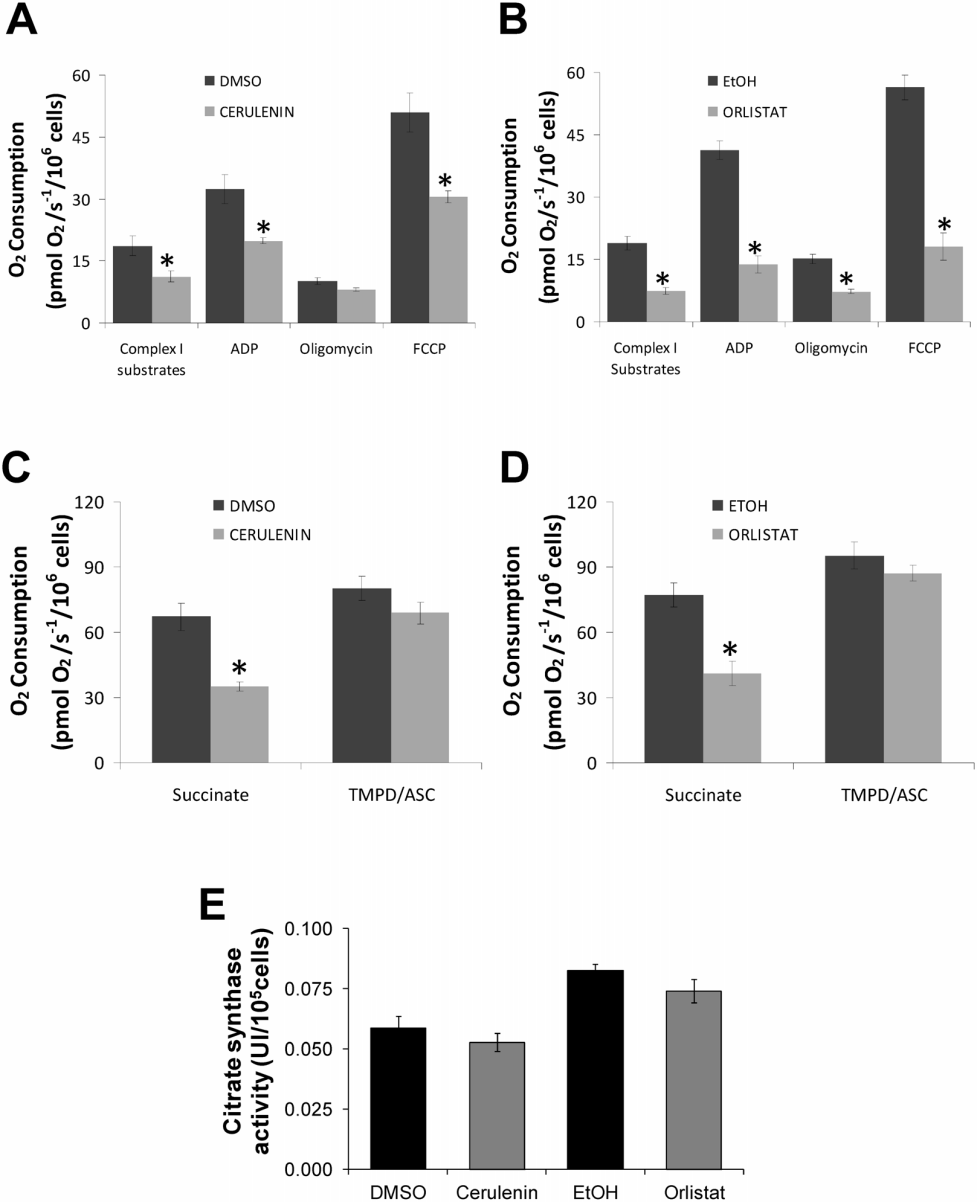
Treatment with FASN inhibitors promotes the inhibition of respiration in melan-a cells. Oxygen consumption by the melan-a cells was measured after treatment for 24 h with 22 µM cerulenin (**A**) or for 48 h with 30 µM orlistat (**B**) using high-resolution respirometry (Oroboros) in a closed chamber equipped with a magnetic stirrer and temperature control set to 37°C. Approximately 2×10^6^ viable cells/ml were permeabilized with 15 µM of digitonin and then were added to 2 ml of reaction medium (described in Materials and Methods). Analyses of oxidative phosphorylation and respiratory activity of the mitochondria were made by sequential additions of 300 µM ADP, 2 µg/ml oligomycin, 100 nM carbonylcyanide p-trifluoromethoxyphenylhydrazone (FCCP), 5 mM succinate, 0.5 µM antimycin and 200 µM N,N,N′,N′-tetramethyl-p-phenylenediamine (TMPD) with 2 mM ascorbate (**A–D**). The activity of citrate synthase was measured in melan-a cells after 24 or 48 h of treatment with 22 µM cerulenin or 30 µM orlistat (**E**). The values represent the mean ± s.e.m of at least four independent experiments. *Significantly different from the respective control at *p*<0.05.

*In situ* analysis of the energy-linked functions of melan-a mitochondria indicated that treatment with the FASN inhibitors decreased the ΔΨm and inhibited respiration (**Figures 4** and **5**). Digitonin-permeabilized melan-a cells showed the ability to phosphorylate ADP, as illustrated by the carboxyatractyloside (CAT) sensitive decrease in ΔΨm (**Figure 4, panels A and B, black lines**), while both the cerulenin and orlistat treatments (**Figure 4, panels A and B, green lines**) resulted in significant decreases in ΔΨm and the inability of the mitochondria to respond to the addition of ADP and CAT.

To gain a better understanding of the possible mechanism involved in mitochondrial dysfunction, we analyzed the mitochondrial generation of superoxide in the melan-a cells. Figure 4C shows a significant increase in mitochondrial superoxide production when melan-a cells were incubated with either FASN inhibitor. Incubation of the cells with the antioxidant N-acetylcysteine (NAC) prevents the increased superoxide generation promoted by these FASN inhibitors. Because mitochondria can be both an important source and target of ROS [73], we then analyzed the possible mitochondrial dysfunctions caused by ROS attack. Therefore, the nature of the ΔΨm decrease by FASN inhibitors was assessed by measuring mitochondrial respiration under both uncoupled or phosphorylating conditions using NADH-linked substrates, succinate or the complex IV substrate TMPD (**Figure 5**). The results presented in **panels A** and **B** show that both cerulenin and orlistat resulted in a significant inhibition of NADH-linked substrate-supported respiration. As expected, the extent of the respiration inhibition was more significant at the maximum rates (FCCP present). P**anels C** and **D** show the rates of uncoupled succinate or TMPD-supported respiration, which indicate a significant inhibition of succinate but not of TMPD oxidation. **Panel E** provides evidence that the FASN inhibitors did not alter the number or mass of mitochondria, as indicated by the lack of an observable effect on citrate synthase activity. Therefore, our results show that the FASN inhibitors activate apoptosis in melan-a cells through a mechanism that involves the inhibition of mitochondrial respiration.

**Figure 6:**
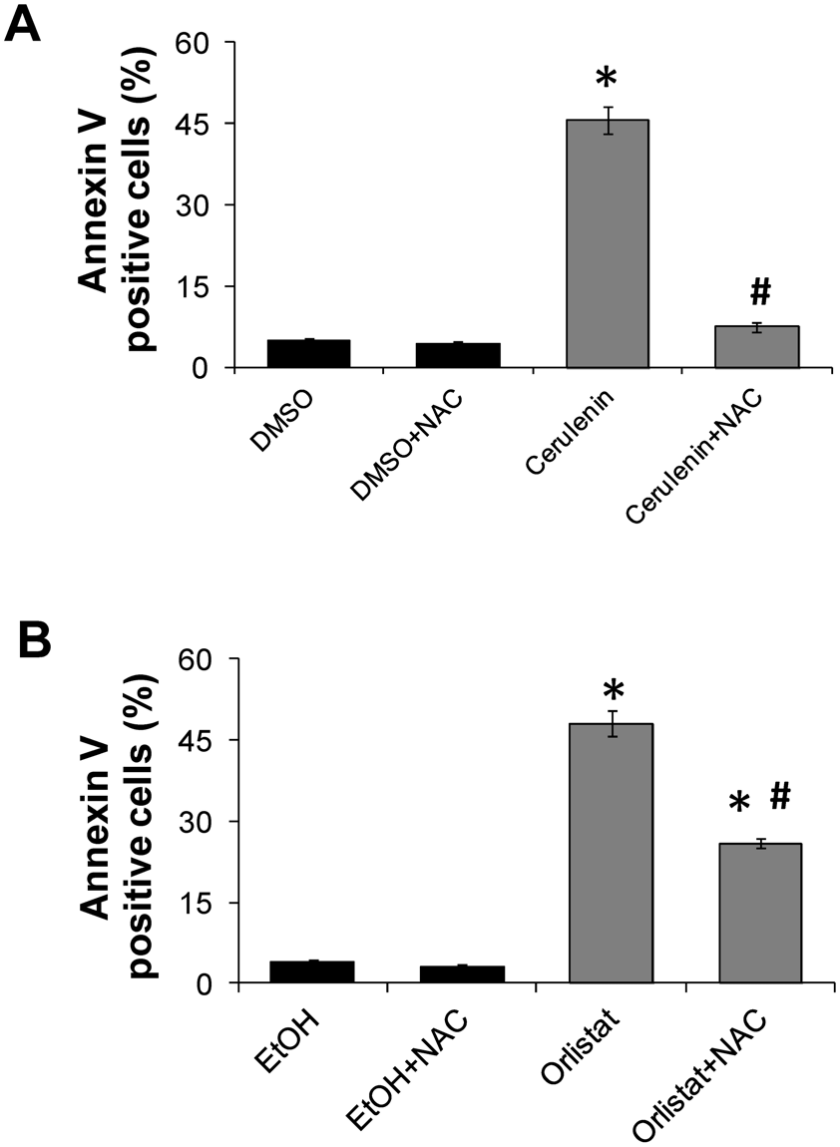
NAC pre-incubation protects melan-a cells from cerulenin or orlistat-induced apoptosis. Melan-a cells were pre-incubated with 5 mM NAC for 1 h, followed by treatment with 22 µM cerulenin for an additional 24 h (**A**) or 30 µM orlistat for 48 h (**B**). NAC was also present during the incubations with cerulenin or orlistat. Apoptosis was then determined by flow cytometry after Annexin V staining. The values represent the mean ± s.e.m of six independent experiments. *Significantly different from the respective control at *p*<0.05. #Significantly different from the respective condition in the absence of NAC at *p*<0.05.

To determine whether FASN inhibitor-induced cell death can be prevented by ROS scavenging agents, further experiments were conducted in the presence of N-acetyl cysteine (NAC). NAC produces important metabolic products that control the cellular redox state, protect cells against mitochondrial dysfunctions associated with oxidative stress, and act as ROS scavengers [74]–[77]. Melan-a cells were then incubated with 5 mM NAC for 1 h prior to cerulenin or orlistat treatment. The results show that the melan-a cell death induced by cerulenin and orlistat were inhibited by 87% and 47%, respectively, in the presence of NAC (**Figure 6, A and B**).

### Cerulenin- and orlistat-induced apoptosis events in melan-a type cells occur independently of FASN inhibition

**Figure 7:**
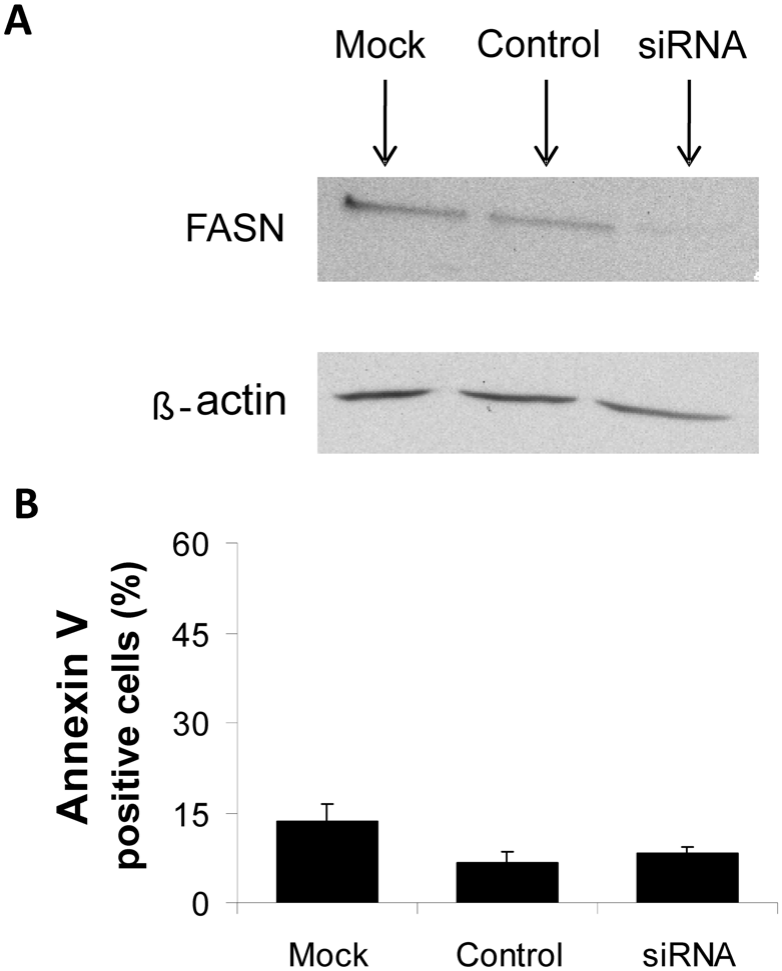
FASN silencing does not induce apoptosis in melan-a cells. Melan-a cells were either transfected with specific siRNAs (siRNA), treated with the transfection reagent alone (mock) or maintained in culture with equimolar concentrations of a nonspecific control oligo (control). Cells were incubated for 48 h. FASN protein content was determined by Western blot analysis (**A**), and apoptosis was estimated by flow cytometry after Annexin V staining (**B**). The values represent the mean ± s.e.m of three independent experiments.

To determine whether the effects of cerulenin and orlistat on cell viability, proliferation and mitochondrial function were related to the actions of the respective agents on FASN, the enzymatic activity of FASN was evaluated. Despite the detection of FASN protein by Western blot analysis (**Figure S2**), the enzymatic activity of FASN in melan-a cells was too low for quantification using radioactive markers with higher sensitivity, such as [^3^H]-water and [^14^C]-acetate *(data not shown*), as was previously performed for B16-F10 melanoma cells [46]. These results were supported by siRNA experiments that successfully down-regulated FASN expression in melan-a cells by significantly reducing protein levels (**Figure 7 A**) without a corresponding increase in apoptotic rates (**Figure 7 B**).

ESI-MS was performed in melan-a cells to analyze the mitochondrial FFA composition after treatment with the FASN inhibitors. In contrast to what we observed in the melanoma B16-F10 cells [49], the incubation of the non-tumorigenic cells with cerulenin or orlistat did not significantly alter the FFA content when principal component analysis (PCA) was applied to the ESI-MS data. The relative concentration (%) of palmitic acid was not significantly modified after treatment with the FASN inhibitors (DMSO and cerulenin: 38.6 and 43.4%, respectively; EtOH and orlistat: 41.6 and 39.0%, respectively). Additionally, non-significant alterations were observed for the most abundant FFAs detected, which include myristic acid (DMSO and cerulenin: 15.0 and 10.2%, ethanol and orlistat: 20.1 and 24.4%, respectively) and stearic acid (DMSO and cerulenin: 26.6 and 31.8%, EtOH and orlistat: 22.1 and 14.5%, respectively). Together, the ESI-MS and RNAi results suggest that the effects of cerulenin and orlistat on melan-a proliferation and viability are independent of their effects on FASN levels and activity.

## Discussion

FASN has been described as a possible target for chemotherapy because its expression is low or absent in most normal tissues and, in contrast, is high in a significant variety of human malignant tumors, where FASN plays important roles in proliferation [10], [12], [13], [16], [18]–[20], [22]–[25], [27], [28], [30]–[32]. As a result, FASN inhibitors are potential antitumor agents due to their ability to reduce cell proliferation and induce apoptosis [44], [47], [57], [78] without apparent toxicity to normal tissues [50], [51]. Although the role of FASN in nonmalignant cells remains uncertain, it is known that the FASN inhibitor cerulenin promotes a reduction in the proliferation of normal fibroblasts in primary cultures [52], [53], and orlistat has been shown to have antiproliferative effects in human umbilical vein endothelial cells (HUVEC) [26], [48], [54].

Here, we analyzed the mechanisms of toxicity of the FASN inhibitors cerulenin and orlistat in cells derived from non-tumorigenic mouse melanoblasts. When these melan-a cells were incubated with FASN inhibitors, the cells underwent apoptosis and exhibited a reduced proliferative rate (**Figure 1**), an increased percentage of cells in G0/G1 phase (**Figure 2 A**) and an increased level of p21^WAF1/Cip1^ tumor suppressor protein (**Figure 2 B**). We also evaluated the effects of the FASN inhibitors on non-tumorigenic HaCaT cells. Although these cells were more resistant to apoptosis (**Figure S1**) than the melan-a cells (**Figure 1**), the HaCaT cells underwent a significant cell cycle arrest (**Figures S3**). These results are in agreement with data showing that FASN inhibitors promote cell death and cell cycle arrest, along with the increased expression of both p21^WAF1/Cip1^ and p53 in colon, breast, gastrointestinal and human melanoma tumor cells [45], [78]–[80].

The increased number of annexin V-positive melan-a cells (**Figure 1 E and F**), the release of cytochrome c (**Figure 3 A**) and the activation of caspases-9 and -3 (**Figure 3 B and C**) are compatible with the activation of the intrinsic apoptosis pathway [81]–[83]. Similar results have been obtained with neuroblastoma, breast cancer and melanoma cell lines [37], [44], [47]. This interpretation is also supported by the lack of caspase-8 activation by the FASN inhibitors, a result that excludes the extrinsic apoptosis pathway in melan-a cell death [84]. In agreement with our results, the exclusion of the extrinsic apoptosis pathway was previously reported for breast tumor cells after FASN inhibition [42]. Additionally, the increased expression of p21^WAF1/Cip1^ (**Figure 2 B** **and Figure S3 B**) suggests that p53 was activated when melan-a and HaCaT cells were treated with cerulenin or orlistat. However, the present data suggest that the FASN inhibitor-induced apoptosis was independent of the activation of p53 in the melan-a cells because pre-treatment with pifithrin-alpha (PFT) [72] did not prevent cell death (**Figure S4 B**). These data are supported by other results, indicating that p53 did not participate in cerulenin-induced cell death in neuroblastoma, melanoma, colon carcinoma, breast cancer, skin carcinoma and glioma tumor cells [44], [47], [57], [80]. Accordingly, cerulenin toxicity was higher in p53 knockout cells than in control RKO colon carcinoma cells [45].

To further investigate the role of FASN activity in nonmalignant cells, we successfully down-regulated FASN expression in melan-a cells using siRNA (**Figure 7 A**) and showed that the apoptosis rate remained unchanged (**Figure 7 B**). Additionally, using the ESI-MS technique, we also demonstrated that the mitochondrial FFA content and composition did not change. Taken together, the siRNA and ESI-MS results suggest that the FASN inhibitors act on melan-a cell mitochondria independently of the changes in FASN activity.

To uncover the link between mitochondrial dysfunction and apoptosis in melan-a cells treated with the FASN inhibitors, we analyzed the mitochondrial energy-linked functions in these cells. Both cerulenin and orlistat were able to independently inhibit the rates of NADH-linked and succinate-supported respiration. This result was followed by both a decrease in ΔΨm (**Figure 4 A and B**) and a significant increase in superoxide production (**Figure 4 C**). In agreement with these results, it was previously reported that silencing the ACC-α (acetyl-CoA carboxylase α) and FASN genes resulted in increased ROS production and mitochondrial dysfunction [85].

The lack of change in both the rate of respiration supported by the complex IV substrate TMPD (**Figure 5 E**) and the activity of citrate synthase provides evidence that treatment with the FASN inhibitors did not alter either the number of mitochondria or the mitochondrial mass (**Figure 5 F**). Therefore, we hypothesize that the decreased respiration rates are the consequence of damage to respiratory complexes I and II. It is well known that the inhibition of these respiratory complexes by various metabolic inhibitors, including statins [66], [86], is mediated by superoxide anion attack on the 4Fe-4S clusters [87]. The results indicating that NAC pre-incubation prior to treatment with FASN inhibitors significantly protected the melan-a cells from apoptosis (**Figure 6 A and B**) support the interpretation that the mitochondrial dysfunction observed here is the consequence of superoxide attack to respiratory complexes I and II. Additionally, it has been reported that cell death *via* the activation of the intrinsic apoptosis pathway can further stimulate ROS production and disrupt respiratory chain complexes I and II through caspase 3, as discussed below.

During apoptosis, the mitochondrial outer membrane becomes permeable to pro-apoptotic proteins, such as cytochrome c, which lead to the formation of apoptosome complexes through the interaction of caspase-9 and APAF-1 [88]. The activation of downstream caspase-3 results in the cleavage of specific substrates that induce DNA fragmentation, nuclear condensation, phosphatidylserine externalization and membrane blebbing [89]–[93]. Further, caspase-3 activation inhibits respiration at the levels of complexes I and II [94]. NADH dehydrogenase Fe-S protein 1 (NDUFS1 or p 75), which is the largest subunit of complex I, serves as a substrate for the activity of caspase-3 during apoptosis. Cleavage by p75 directly inhibits the function of complex I, leading to ΔΨm collapse, ROS generation and mitochondria damage [95].

In conclusion, the present results indicate that the FASN inhibitors cerulenin and orlistat induced apoptotic death in the non-tumorigenic cell line melan-a through a mechanism associated with the activation of the intrinsic apoptotic pathway, mitochondrial oxidative stress and respiratory chain impairment, independent of FASN inhibition.

## Supporting Information

Figure S1 **Cerulenin and orlistat reduce cell viability and induce apoptosis in the HaCaT cell line.** HaCaT cells were treated with increasing concentrations of cerulenin or orlistat for 24 or 48 h, respectively; cell viability was determined using trypan blue (**A** and **B**) or MTT assays (**C** and **D**), and apoptosis was determined by flow cytometry (**E** and **F**). The values represent the mean ± s.e.m of at least three independent experiments. *Significantly different from the respective control at *p*<0.05.
(TIF)

Figure S2 **The fraction of FASN protein is higher in melan-a than HaCaT cells.** Equal amounts of total protein (40 µg) were electrophoretically separated, and the membranes were incubated with antibodies against FASN or beta-actin. Western blot analysis showed that the FASN content was 2.3-fold higher in the melan-a cells than in the HaCaT cells (0.678 *versus* 0.294 a.u., melan-a *versus* HaCaT; data normalized using beta-actin).
(TIF)

Figure S3 **FASN inhibitors blocked cell cycle progression in non-tumorigenic cells.** HaCaT cells (**A**) were treated with 45 µM cerulenin or 300 µM orlistat for 24 or 48 h, respectively. Then, the percentage of cells in each phase of the cell cycle was determined by flow cytometry after PI staining. Western blot analysis of the protein extracts prepared from cerulenin- and orlistat-treated HaCaT cells revealed the accumulation of p21WAF1/Cip1 tumor suppressor protein; the data were normalized using beta-actin as a loading control (**B**). The values represent the mean ± s.e.m of at least five independent experiments. *Significantly different from the respective control at *p*<0.05.
(TIF)

Figure S4 **FASN inhibitor-induced apoptosis is independent of mitochondrial permeability transition or p53 in melan-a cells.** Melan-a cells were treated with 22 µM cerulenin or 30 µM orlistat for 24 or 48 h, respectively, in the presence of cyclosporin A (CsA, 1 µM) (**A**) or (**B**) pifithrin-alpha (PFT, 10 µM); then, apoptosis was determined by flow cytometry after Annexin V staining. The values represent the mean ± s.e.m of five independent experiments. *Significantly different from the respective control at *p*<0.05.
(TIF)
