# Supplementary figures and images for: Fatty Acid Synthase Inhibitors Induce Apoptosis in Non-Tumorigenic Melan-A Cells Associated with Inhibition of Mitochondrial Respiration
Source: PLoS One. 2014 Jun 25;9(6):e101060. doi: 10.1371/journal.pone.0101060 (PMC4071076; doi:10.1371/journal.pone.0101060)

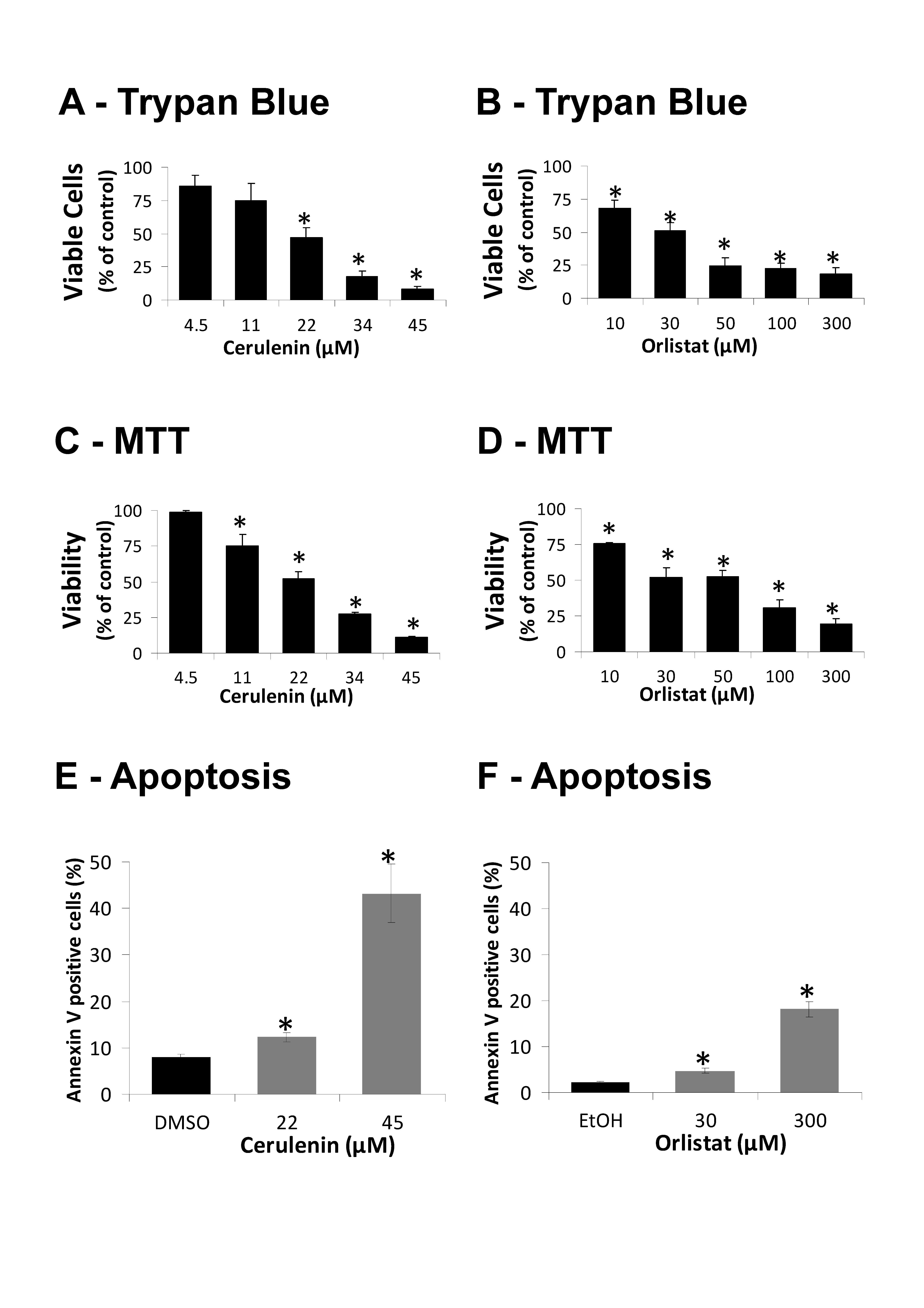

Supplement: Figure S1 — Cerulenin and orlistat reduce cell viability and induce apoptosis in the HaCaT cell line. HaCaT cells were treated with increasing concentrations of cerulenin or orlistat for 24 or 48 h, respectively; cell viability was determined using trypan blue (A and B) or MTT assays (C and D), and apoptosis was determined by flow cytometry (E and F). The values represent the mean ± s.e.m of at least three independent experiments. *Significantly different from the respective control at p<0.05. (TIF) [file pone.0101060.s001.tif]

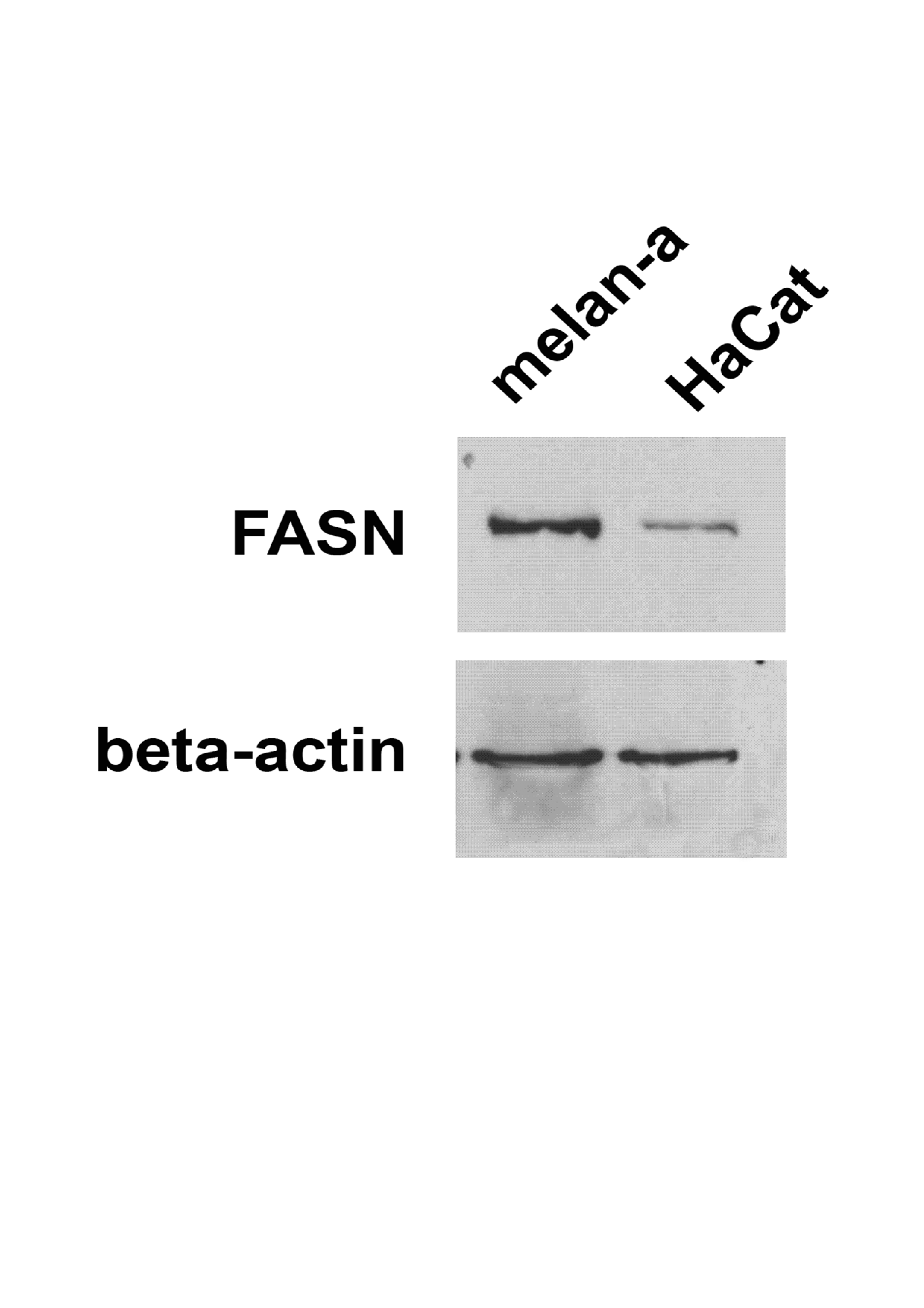

Supplement: Figure S2 — The fraction of FASN protein is higher in melan-a than HaCaT cells. Equal amounts of total protein (40 µg) were electrophoretically separated, and the membranes were incubated with antibodies against FASN or beta-actin. Western blot analysis showed that the FASN content was 2.3-fold higher in the melan-a cells than in the HaCaT cells (0.678 versus 0.294 a.u., melan-a versus HaCaT; data normalized using beta-actin). (TIF) [file pone.0101060.s002.tif]

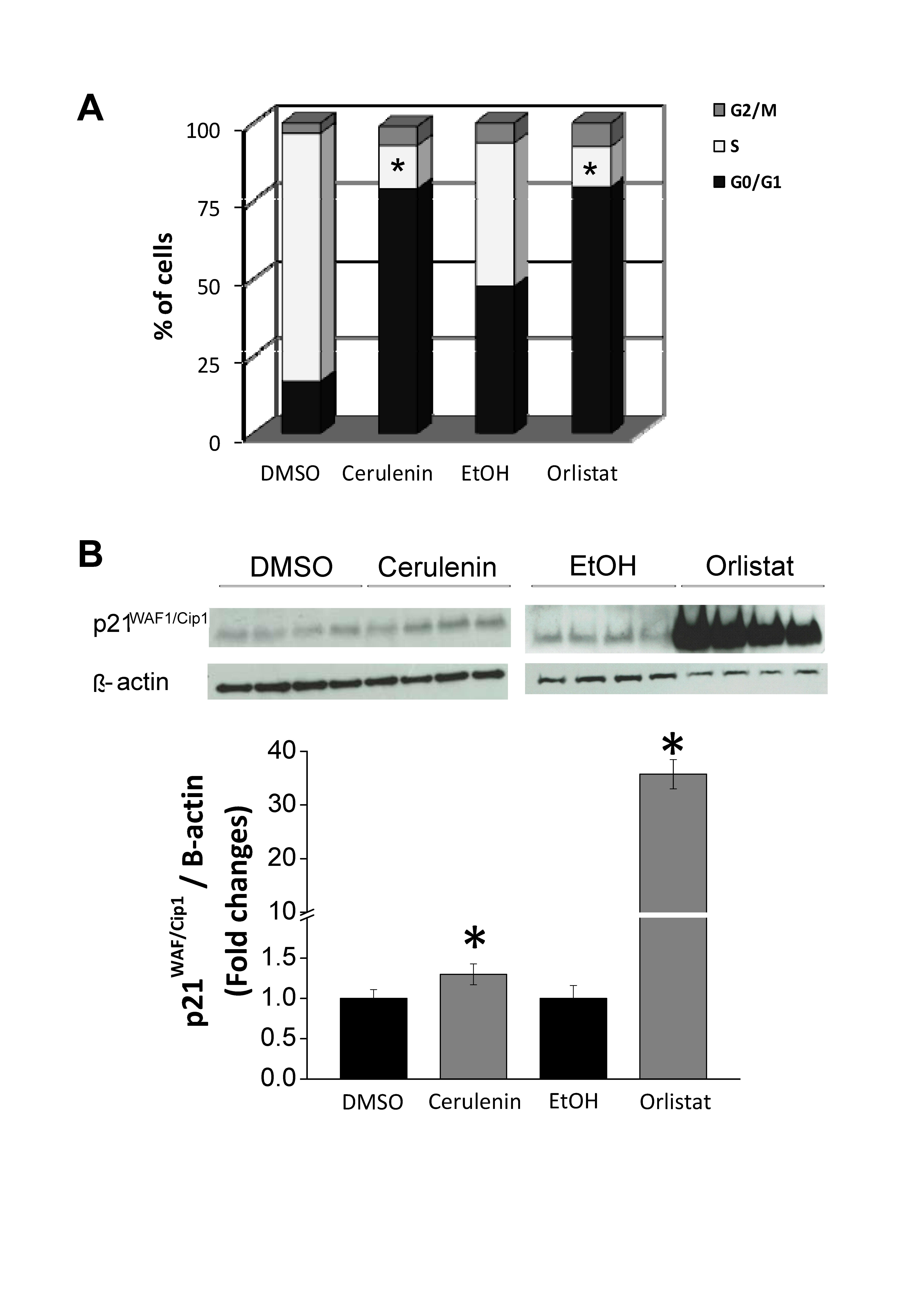

Supplement: Figure S3 — FASN inhibitors blocked cell cycle progression in non-tumorigenic cells. HaCaT cells (A) were treated with 45 µM cerulenin or 300 µM orlistat for 24 or 48 h, respectively. Then, the percentage of cells in each phase of the cell cycle was determined by flow cytometry after PI staining. Western blot analysis of the protein extracts prepared from cerulenin- and orlistat-treated HaCaT cells revealed the accumulation of p21WAF1/Cip1 tumor suppressor protein; the data were normalized using beta-actin as a loading control (B). The values represent the mean ± s.e.m of at least five independent experiments. *Significantly different from the respective control at p<0.05. (TIF) [file pone.0101060.s003.tif]

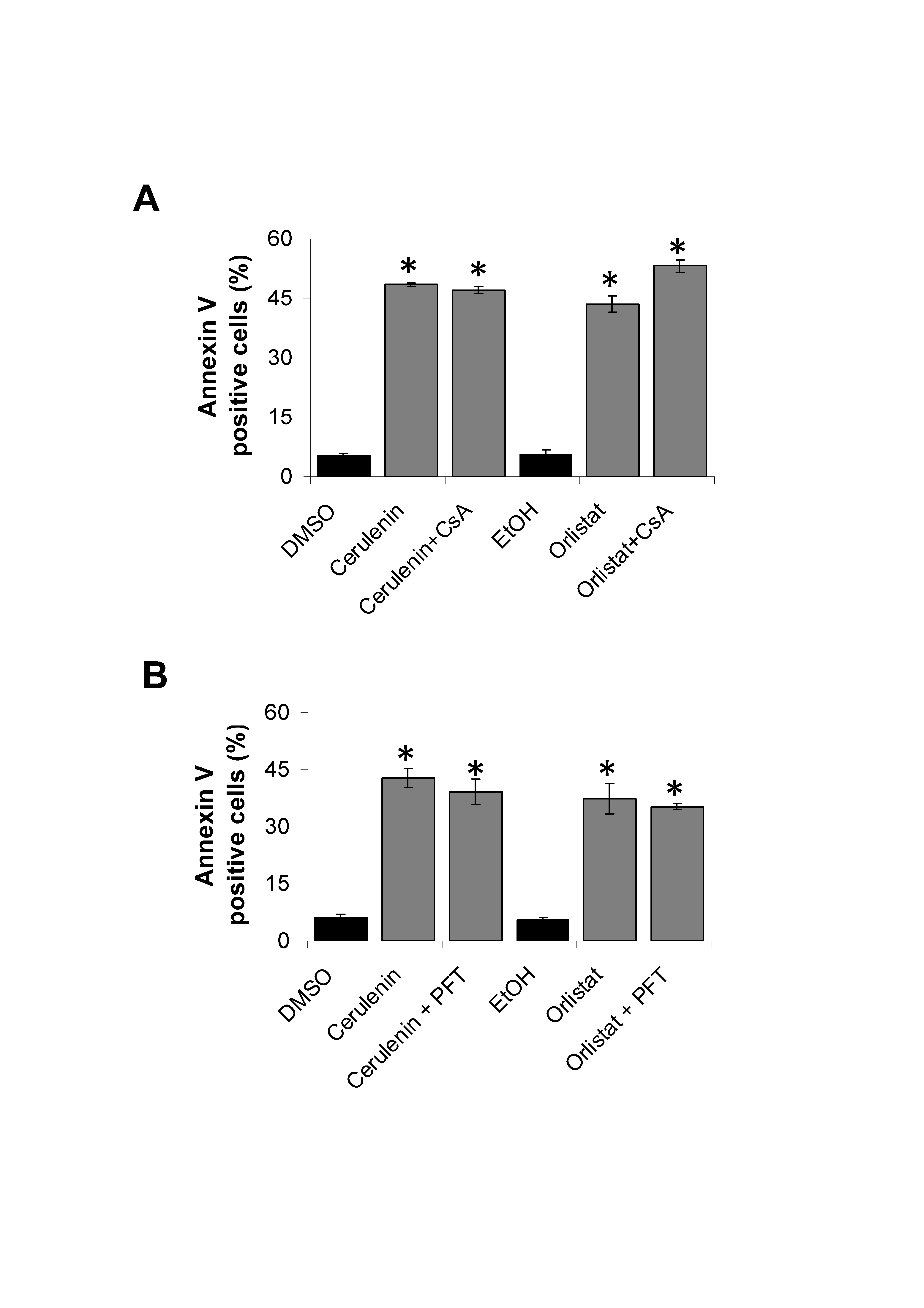

Supplement: Figure S4 — FASN inhibitor-induced apoptosis is independent of mitochondrial permeability transition or p53 in melan-a cells. Melan-a cells were treated with 22 µM cerulenin or 30 µM orlistat for 24 or 48 h, respectively, in the presence of cyclosporin A (CsA, 1 µM) (A) or (B) pifithrin-alpha (PFT, 10 µM); then, apoptosis was determined by flow cytometry after Annexin V staining. The values represent the mean ± s.e.m of five independent experiments. *Significantly different from the respective control at p<0.05. (TIF) [file pone.0101060.s004.tif]
